# Balancing reality in embedded research and evaluation: Low vs high embeddedness

**DOI:** 10.1002/lrh2.10294

**Published:** 2021-11-03

**Authors:** George L. Jackson, Laura J. Damschroder, Brandolyn S. White, Blake Henderson, Ryan J. Vega, Amy M. Kilbourne, Sarah L. Cutrona

**Affiliations:** ^1^ Center of Innovation to Accelerate Discovery and Practice Transformation (ADAPT) Durham VA Health Care System Durham North Carolina USA; ^2^ Department of Population Health Sciences Duke University Durham North Carolina USA; ^3^ Division of General Internal Medicine, Department of Medicine Duke University Durham North Carolina USA; ^4^ Department of Family Medicine and Community Health Duke University Durham North Carolina USA; ^5^ Center for Clinical Management Research VA Ann Arbor Healthcare System Ann Arbor Michigan USA; ^6^ Office of Healthcare Innovation and Learning United States Veterans Health Administration Washington District of Columbia USA; ^7^ Quality Enhancement Research Initiative (QUERI) United States Veterans Health Administration Washington District of Columbia USA; ^8^ Department of Learning Health Sciences University of Michigan Ann Arbor Michigan USA; ^9^ Center for Healthcare Organization & Implementation Research Bedford & Boston VA Medical Centers Bedford Massachusetts USA; ^10^ Division of Health Informatics and Implementation Science, Department of Population and Quantitative Health Sciences University of Massachusetts Medical School Worcester Massachusetts USA; ^11^ Division of General Internal Medicine, Department of Medicine University of Massachusetts Medical School Worcester Massachusetts USA

**Keywords:** diffusion, embedded research, implementation science, Quality Enhancement Research Initiative (QUERI), veterans

## Abstract

Embedding research and evaluation into organizations is one way to generate “practice‐based” evidence needed to accelerate implementation of evidence‐based innovations within learning health systems. Organizations and researchers/evaluators vary greatly in how they structure and operationalize these collaborations. One key aspect is the degree of embeddedness: from low embeddedness where researchers/evaluators are located outside organizations (eg, outside evaluation consultants) to high embeddedness where researchers/evaluators are employed by organizations and thus more deeply involved in program evolution and operations. Pros and cons related to the degree of embeddedness (low vs high) must be balanced when developing these relationships. We reflect on this process within the context of an embedded, mixed‐methods evaluation of the Veterans Health Administration (VHA) Diffusion of Excellence (DoE) program. Considerations that must be balanced include: (a) low vs high alignment of goals; (b) low vs high involvement in strategic planning; (c) observing what is happening vs being integrally involved with programmatic activities; (d) reporting findings at the project's end vs providing iterative findings and recommendations that contribute to program evolution; and (e) adhering to predetermined aims vs adapting aims in response to evolving partner needs.

## 1 INTRODUCTION

Embedded research and evaluation programs link rigorous scientific processes and research methods with clinical, business, and operational needs in a healthcare organization.^1^, ^2^ This type of partnered linkage is important to the process of implementation science, and allows healthcare systems to function as learning health systems, balancing scientific expertise with quality improvement perspectives in order to identify, evaluate, and implement innovative practices.^3^, ^4^, ^5^ An embedded research/evaluation relationship benefits both the healthcare organization and the research/evaluation team. Organizations profit from the methodologic rigor and expertise of research/evaluation teams while producing evidence relevant to their own context and circumstances. Generated evidence is ready for rapid application to operational needs while the research/evaluation team can have a rapid impact on the services provided to patients.

While embedded research and evaluation has received increased attention in recent years,^6^, ^7^ the concept of “embeddedness” has been described in organizational research for over 70 years. Key concepts include the goals leading to embedded relationships, the structures upholding and strengthening these relationships, and the need to develop trust between members of groups who come to the relationship from differing institutional cultures. Additional areas of focus include the process by which organizations and groups learn from each other and maintenance of embedded relationships over time.^8^

The potential for embedded research/evaluation has received increasing attention across fields such as education and healthcare.^6^, ^9^ The focus on close collaboration between researchers/evaluators and operational leaders within organizations is still an evolving area, and the embedded research/evaluation relationships take many forms. Researchers and evaluators may have a low degree of embeddedness, with roles akin to outside consultants, or they may be highly involved in both program evaluation and operations alongside other health system staff, a relationship of high embeddedness. Many embedded relationships fall somewhere in between. Conscious reflection (by researchers/evaluators and their organizations) on the degree of research team embeddedness is essential to effectively plan roles and to recognize capacities and limitations of the research/evaluation team.^1^

Although the Department of Veterans Affairs (VA) has a long history of embedded research and evaluation through a robust Health Services Research & Development (HSR&D) program and the Quality Enhancement Research Initiative (QUERI),^3^, ^10^, ^11^ there are challenges in maintaining the objectivity demanded of bias‐free research or evaluation. In this paper, we present lessons learned from our embedded evaluation experience. We use the experience of conducting the evaluation of the Veterans Health Administration (VHA) Diffusion of Excellence (DoE) program as a basis for demonstrating lessons that can be considered by others who may embark on an embedded research or evaluation project in partnership with non‐research health system colleagues. We end with a set of lessons to consider when conducting embedded projects.

## 2 CONTEXT‐DIFFUSION OF EXCELLENCE PROGRAM

In October 2015, the Department of Veterans Affairs (VA) Under Secretary for Health initiated the VHA Diffusion of Excellence (DoE) program. DoE has progressed to become an integral part of the broader Innovation Ecosystem in the VHA. The objectives of the DoE program are to: (a) identify promising clinical and administrative practices successfully developed and tested by individuals at the frontline of care; (b) pilot these promising practices in new facilities and provide implementation facilitation support for implementing teams; and (c) support the scale‐up and spread of successful practices across the health system.^12^, ^13^, ^14^, ^15^, ^16^, ^17^

DoE is a nationwide program that employs implementation strategies for promoting adoption, implementation, and spread of promising practices across VHA. Frontline staff members apply for the chance to pitch their practices during a national “Shark Tank” competition; VHA facility directors serve as “Sharks” who place bids. The winning practices are designated DoE Promising Practices. Winning Shark bidders receive 6 to 9 months of external implementation consultation at their facility. DoE then provides national support for diffusing successful practices across the VHA in cooperation with national program offices. Diffusion support may take one of three different paths, including: (a) direct facilitation by DoE staff for a small number of DoE National Diffusion Practices; (b) initial assistance with diffusion and training of practice developers through a VHA Diffusion Academy for a second set of DoE Promising Practices; or (c) promotion of organic diffusion across the VHA through packaged information for the final group of DoE Promising Practices.

The DoE program has also developed an online Diffusion Marketplace that provides information about these practices so that facilities seeking to address specific challenges can search the Diffusion Marketplace for potential solutions that have shown positive impact in VHA. The Diffusion Marketplace also helps to track diffusion and implementation of practices across the VHA.^15^

DoE has had broad reach in the VHA. Over the course of six Shark Tanks, there have been 2671 applications from across the VHA, which has led to the designation of 69 DoE Promising Practices. As of the end of fiscal year 2020, DoE estimates that eight National Diffusion Practices have been implemented at numerous facilities across the VHA and impacted an estimated 570 000 veterans in fiscal years 2019 and 2020. Example DoE Promising Practices include (a) enhancing oral hygiene of inpatients with the goal of preventing non‐venerator associated pneumonia^18^, ^19^; (b) encouraging the early ambulation of hospitalized patients with the goal of preventing functional decline^20^, ^21^, ^22^; and (c) use of automated review of electronic health record data to identify prescriptions that are potential candidates for discontinuation.^23^

## 3 EMBEDDED EVALUATORS WITH THE SPREADING HEALTHCARE ACCESS, ACTIVITIES, RESEARCH, AND KNOWLEDGE (SHAARK) PARTNERED EVALUATION INITIATIVE

At an early stage of program development, DoE leaders recognized the benefits of rigorous evaluation to describe and measure the program's reach, effectiveness, adoption, implementation, and maintenance (ie, components of the RE‐AIM evaluation framework).^24^, ^25^ To obtain this evaluation, DoE partnered with a multidisciplinary team of QUERI‐funded evaluators selected through a competitive national peer‐review grant process. The evaluation team is conducting an embedded mixed‐methods evaluation of the DoE called the Spreading Healthcare Access, Activities, Research, and Knowledge (SHAARK) Partnered Evaluation Initiative. The ongoing evaluation is guided by implementation science frameworks and theories including the consolidated framework for implementation research (CFIR),^26^ theory of organizational readiness for change,^27^ and theory of diffusion of innovation^28^ and uses a variety of qualitative (structured observations, semi‐structured interviews, focus groups) and quantitative data (performance data, systems to track practice implementation, surveys) to triangulate evaluation findings.^14^, ^15^

Per regulations outlined in VHA Program Guide 1200.21, the SHAARK evaluation has been designated a non‐research quality improvement activity. Although QUERI funds teams of research investigators and staff across VHA facilities nationally, these projects are funded using clinical dollars, not as congressionally appropriated research. QUERI funded the initial SHAARK evaluation as well as select “DoE Promising Practices” identified through the Diffusion of Excellence Shark Tank process and by VA national leaders as top priority for national diffusion. Funding for later phases of these evaluations has been shared between QUERI and DoE through funding from the VHA Office of Rural Health (ORH) as well as other national VHA program offices, which provided an indication of the perceived operational value of the evaluation. For this analysis, we report on the experience involving the global evaluation of the DoE program through the national SHAARK evaluation.

Unlike one‐time evaluations of programs (where an external evaluator enters, observes, and emerges with a report on process and impact), the SHAARK team built a long‐term relationship with DoE leaders. Transitions in core DoE leadership and staff have occurred several times over the past years, thus the longevity of the evaluation team's relationship has been particularly valuable. SHAARK team members have worked alongside successive DoE leaders to develop and refine key questions, establish rationale for methods, interpret findings, and iteratively integrate these findings into strategic planning and programing activities. To complete a meaningful evaluation, research team members have balanced responsiveness within a valuable collaborative relationship with the responsibility of maintaining objectivity.

The initial goals of the SHAARK evaluation were developed with original leaders of the DoE program, whose focus was on rapidly standing up a high‐visibility program (the Shark Tank), supporting participants, and institutionalizing the program. Aims focused on explaining program participation, motivations, and processes used for bidding in the Shark Tank, and implementation effectiveness in sites receiving facilitated implementation support as a result of having a “winning” Shark Tank bid.

As described above, DoE has expanded its facilitation role over the past 5 years toward promoting spread of successful innovations across the VHA through a combination of direct staff support, training, and active tracking spread across the healthcare system. As a result, SHAARK has also pivoted from a focus primarily on understanding the Shark Tank and early facilitated replication of innovation toward understanding how to help DoE support innovations once they have completed the initial replication stage. The goal is to consider how to help the VHA as a whole take maximum advantage of DoE Promising Practices as it seeks to serve veterans across the country.

## 4 BALANCING THE DEGREE OF EMBEDDEDNESS

**FIGURE 1:**
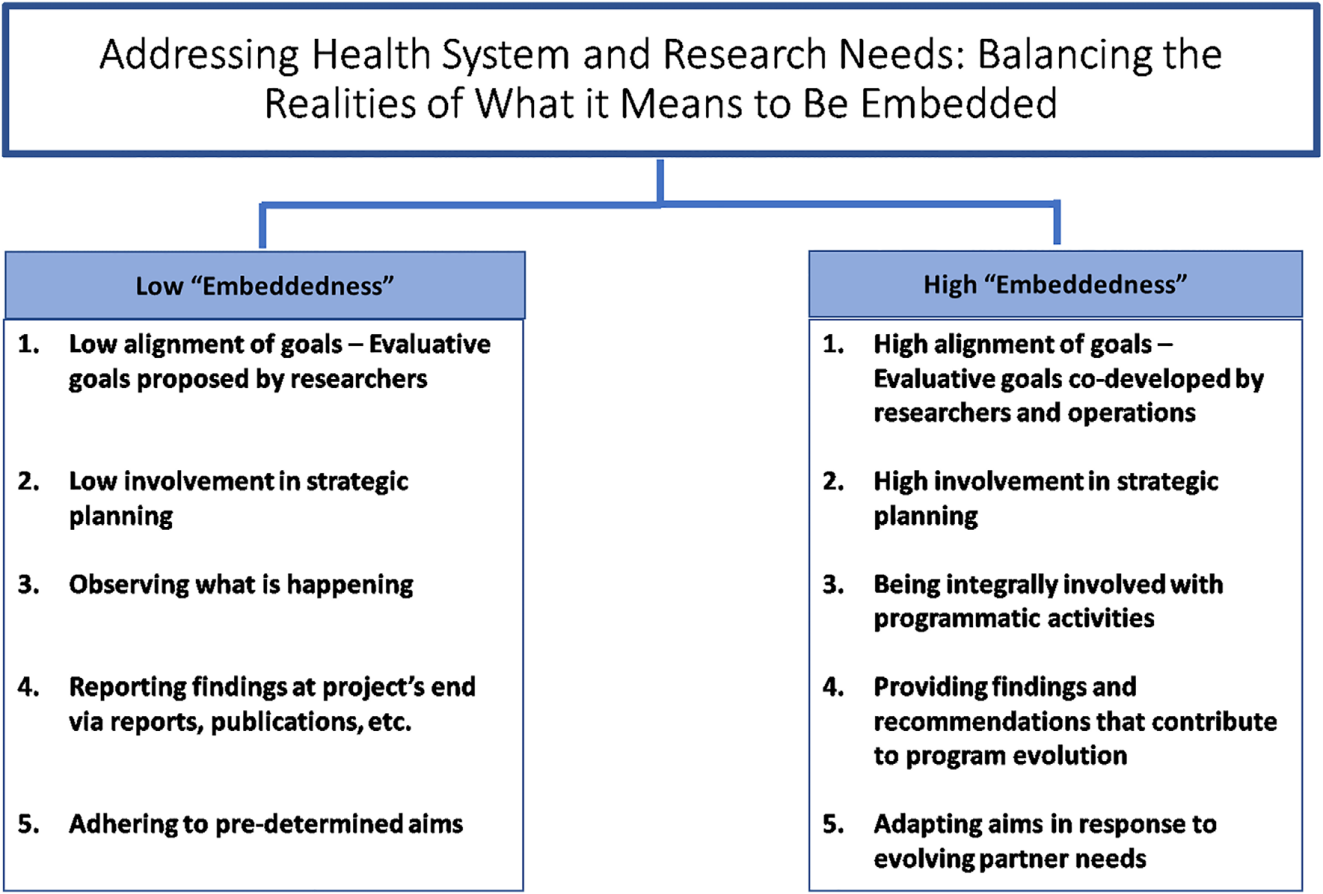
Addressing health system and research needs: Balancing the realities of what it means to be embedded

Below, we discuss considerations that must be balanced in order to maximize the benefits of embedded research or evaluation. Figure 1 shows five key points of balance that were highlighted through our SHAARK team's embedded research. These include: (a) low vs high alignment of goals; (b) low vs high involvement in strategic planning; (c) observing what is happening vs being integrally involved with programmatic activities; (d) reporting findings at the project's end vs providing iterative findings and recommendations that contribute to program evolution; and (e) adhering to predetermined aims vs adapting aims in response to evolving partner needs.

### 4.1 Low vs high alignment of goals

Balancing the alignment of goals required a clear understanding of the goals of the DoE program, without losing sight of the aims of the evaluation itself. Evaluation team members worked to track changing program priorities, to stay connected through leadership changes, and to navigate changing roles and responsibilities assigned to contracted consultants who provide day‐to‐day support for DoE activities. Even as we worked to stay abreast of DoE goals, the evaluation team members maintained a distinct identity, seeking to contribute expertise in implementation science where applicable. Evaluation team members supported close collaboration between DoE and the national QUERI office and served as faculty for DoE events that highlighted the links between implementation science and quality improvement.

### 4.2 Low vs high involvement in strategic planning

SHAARK evaluation findings have informed strategic planning, influencing the evolution of DoE. Early in the evaluation timeline, the SHAARK team found that key DoE participants including Sharks (VHA medical center and regional directors), practice developers, and pilot implementation teams lacked a clear understanding of their roles and expectations for others within DoE. In response, DoE leaders, in collaboration with the SHAARK evaluation team, drafted descriptions of roles and responsibilities that were shared with participants and participation agreements with sites receiving facilitated replication of DoE Promising Practices, leading to programmatically important indication of greater understanding of roles in the DoE process.

### 4.3 Observing what is happening vs being integrally involved with programmatic activities

The SHAARK team has had to navigate the appropriate level of involvement with programmatic aspects of DoE. Through structured observations and semi‐structured interviews, evaluators identified challenges experienced by some local implementation teams that could be traced to poor fit of the practice with local conditions.^14^ To help address this challenge, the SHAARK team developed a “QuickView” tool to provide easy‐to‐read summaries and practice comparisons for the finalist practices in the Shark Tank. This tool uses simple, engaging graphics and color coding with easily understandable icons to help guide Shark bid preparations. The “QuickView” was emailed to Sharks nationwide, before the Shark Tank and hardcopies were broadly available during the Shark Tank. Following positive reception of the “QuickView,” a second document, a bid “Wish List” was developed that clearly lays out what needs to be in place at facilities to maximize the likelihood of successful practice implementation. These documents went from being a product of SHAARK to being fully integrated into DoE operations, an example in which implementation science expertise was applied to the development (and assessment of impact) of a pragmatic tool.

### 4.4 Reporting findings at the project's end vs providing iterative findings and recommendations that contribute to program evolution

The evaluation team's focus on building strong partnerships with stakeholders such as DoE leadership, VHA program offices, VHA facility leaders, and participants in DoE programs has prompted the delivery of frequent reports, recommendations, and discussions. As illustrated above, the provision of iterative findings has influenced DoE's evolution far more than would have been expected from an evaluation that provided a single report after several years' study.

### 4.5 Adhering to predetermined aims vs adapting aims in response to evolving partner needs

There has been continued negotiation as to whether and how evaluation aims should evolve. The partnered nature of the work inevitably leads to changes. To account for these changes, the SHAARK team has renegotiated project scope within available resources with the goal of more fully addressing both the changing nature of the DoE structure and needs of new leadership for specific types of information. Allowing aims to evolve over time has been an effective way to accommodate the needs of the DoE partners, but also poses challenges for publishing evaluation findings within traditional scientific structures (eg, journal manuscripts, academic conference presentations). The non‐research/quality improvement designation of this evaluation enabled a formative approach where subsequent aims were added based on findings from earlier aims and feedback to partners.

## 5 LESSONS LEARNED WHEN BALANCING HIGH VS LOW EMBEDDEDNESS

The SHAARK evaluation team has worked to clarify roles and expectations between the embedded evaluation team and DoE partners while recognizing that we have different perspectives and varying incentives. This is challenging work that takes frequent communication and negotiation. The process has included: frequently meeting formally and informally in‐person (including multiple day‐long planning meetings) to build mutual understanding and trust; evaluators shadowing in‐person and virtual DoE events; regular (bi‐weekly) conference calls with program leaders and contracted consultants; open discussion of both programmatic and evaluation activities; and rapidly addressing any miscommunication or need for clarification.

In many ways, this is the reality of any lasting relationship of any kind where the parties are not required to participate. SHAARK has had an initial grant and two subsequent extensions. Ultimately the reason we continue to work together is that there is an opportunity to help the VA enhance innovations that can positively impact veterans' lives while enjoying the process of working together. Through this process, we have summarized a number of lessons that have allowed for the calibration of the degree of embeddedness. These lessons, which are described in more detail in Table 1, include the need to: recognize that aims may change; negotiate appropriate and reasonable expectations; describe how embedded research/evaluation compares to other options; articulate the added value to all partners (operational and researchers/evaluators); ensure organizational and individual incentives align; and have open exchange of feedback.

**TABLE 1:** Lessons learned when balancing high vs low embeddedness

Lesson summary	Description of lesson	Examples and I = Insights from the SHAARK Evaluation
Recognize that aims may change	Embedded research/evaluation is not static. While bias can be reduced by following through on initial aims, there are times when aims and methods must be adjusted to maximize the utility of the evaluative effort.	The leadership of DoE has turned over since the beginning of SHAARK, and the program is more mature. As a result, evaluation questions have been renegotiated, moving from a primary focus on the operation of the VHA Shark Tank to a focus on the spread of practices across the VHA.
Negotiate appropriate and reasonable expectations	Care must be taken to ensure that scope is appropriate—flexibility is required to be responsive to partner needs without exceeding the bounds of what is manageable for evaluators and researchers.	Partners regularly discuss how changes in operational needs and evaluation questions may impact needed changes in expectations related to both deliverables and timelines. In other words, if we add one thing, what other thing may need to be given up or delayed.
Describe how embedded research/evaluation compares to other options	The embedded research/evaluation team should be prepared to describe the value they bring compared to other options the health system may have for evaluation or consultation.	The SHAARK team has a close working relationship with the management consultants supporting DoE. This includes clarifying operations, collaborating on information obtained from innovator/projects (eg, survey development/administration), and feeding back results to enhance operations.
Articulate the added value to all partners (operational and researchers/evaluators)	The embedded research/evaluation team should be prepared to explain the ways their work provides value to all partners (eg, identification of strategic partnerships for both groups; evaluation team publications and presentations; positive impact on patients when the program meets with success).	SHAARK has: (a) collected stakeholder feedback and conducted analysis of data related to specific elements of the DoE process with a focus on producing information that can be used to make programmatic adjustments; (b) produced operationally focused reports with information that can be incorporated into briefings provided by DoE to their stakeholder; (c) participated on DoE strategic planning; (d) developed specific products such as the “Quickview” described in this paper, and (e) disseminated results through a variety of presentations and publications so that information on the DoE process can be accessed by different audiences.
Ensure organizational and individual incentives align	Researchers/evaluators and other health system staff face different working realities; needs and timelines can be very different and clear communication is particularly important to ensure mutual understanding.	SHAARK and DoE regularly discuss topics such as follows: (a) how individual job performance and the program's value are evaluated; (b) the specific operational changes that DoE have made as a result of SHAARK findings; (c) short‐ and long‐term information needed to inform the program; and (d) the value of different ways of disseminating findings to both operational partners and broader stakeholders.
Have open exchange of feedback	Success is based on the willingness of all partners to provide and receive feedback in a respectful manner.	SHAARK and DoE teams meet every two weeks to discuss operational and evaluation activities, along with joint goals and meaning of findings.

Abbreviations: DoE, Diffusion of Excellence; SHAARK, Spreading Healthcare Access, Activities, Research, and Knowledge; VHA, Veterans Health Administration.

## 6 CONCLUSION

To be effective, embedded research and evaluation efforts rely on strategic alliances between organizational partners.^29^ Collaboration between researchers and organizational partners is not a new phenomenon, but intentional effort is often required to reach outside one's professional circle. For research/evaluation teams who wish to directly inform healthcare operations, these alliances must be established, cultivated, and sustained. Organizational research literature highlights a number of factors as instrumental in the maintenance of alliances. These include goal alignment, regular communication, joint decision‐making with key stakeholders, relationship management, adaptation within a changing environment, and repeated articulation of the value of the alliance to members of the organization.^30^, ^31^, ^32^ Decisions also need to be made about the degree of embeddedness. Partners in the relationship need to decide where they and their relationship will fall on a continuum from “low” to “high” embeddedness. The answer to this question will impact research/evaluation team tasks and involvement in operations and will influence the productivity of the alliance over time.

The potential rewards of these interorganizational strategic alliances are considerable. Researchers extend their networks to ensure relevance and applicability of their work within a learning healthcare system; operational leaders benefit from researcher's methodologic expertise in addressing operational challenges.

Overall, the embedded research/evaluation approach that we describe here has helped to inform VHA's novel DoE program in ways that benefit VHA and that can be shared with outside systems as well. The strong partnership between the SHAARK evaluation team and DoE leaders has relied in equal parts on dedicated efforts by both parties. Together, evaluators with a research background and operational leaders have achieved this partnership through continuous dialogue, frequent re‐examination of the evaluator's level of objectivity, and careful attention to the balancing that characterizes embedded research. These principles are key to the type of partnered research required of both implementation science and achieving learning health system goals of utilizing research and operational expertise to develop innovative strategies to enhance the health and health care of the people they serve.

## CONFLICT OF INTEREST

Authors have no conflicts to declare. All authors are employees of the United States Department of Veterans Affairs. No authors received compensation for preparation of this manuscript outside of their salaries.
